# Central serous chorioretinopathy and the sclera: what we have learned so far

**DOI:** 10.1007/s10384-024-01101-2

**Published:** 2024-08-16

**Authors:** Hideki Koizumi, Naoya Imanaga, Nobuhiro Terao

**Affiliations:** Department of Ophthalmology, Graduate School of Medicine, University of the Ryukyus, 207 Uehara, Nishihara-cho, Nakagami-gun, Okinawa, 903-0215 Japan

**Keywords:** Central serous chorioretinopathy, Pachychoroid, Sclera, Uveal effusion syndrome, Vortex vein

## Abstract

Central serous chorioretinopathy (CSC) is a common disorder characterized by serous retinal detachment. Several studies using indocyanine green angiography (ICGA) have revealed that choroidal filling delay, choroidal vascular dilation, and choroidal vascular hyperpermeability are the characteristic findings of CSC. These ICGA findings confirm that choroidal circulatory disturbances are the primary factors in the pathogenesis of CSC. With advancements in optical coherence tomography (OCT), choroidal thickness has been found to be significantly greater in eyes with CSC than in normal eyes. Dilated large choroidal vessels reportedly account for the thickened choroid in eyes with CSC. Although many possible mechanisms and risk factors have been suggested, the pathophysiologic features of choroidal circulatory disturbances and choroidal thickening in eyes with CSC have not yet been fully elucidated. Recently, using anterior segment OCT, we proposed that the sclera may induce choroidal circulatory disturbances since CSC eyes have significantly thicker sclera than do normal eyes. This review summarizes updated information on the close relationship between CSC pathogenesis and the sclera.

## Introduction

Central serous chorioretinopathy (CSC) is a common disorder that predominantly affects middle-aged men and is characterized by serous retinal detachment [1]. Although retinal detachment resolves spontaneously in many cases, it often becomes chronic and develops secondary macular neovascularization, resulting in poor prognosis [2]. Indocyanine green angiography (ICGA) has dramatically improved our understanding of the pathogenesis of CSC and has revealed findings characteristic of CSC, such as choroidal filling delay, choroidal vascular dilation, and choroidal vascular hyperpermeability [3–6]. The consensus is that the choroid is the primary site of CSC pathogenesis. Recent advances in optical coherence tomography (OCT) have enabled us to obtain cross-sectional images of the choroid in daily practice, and choroidal thickening, dilation of large choroidal vessels, and thinning of the choriocapillaris are assumed to occur in eyes with CSC [7, 8]. These pathologic states of the choroid form the background for the development of serous retinal detachment. Other diseases with choroidal findings similar to those of CSC include pachychoroid pigment epitheliopathy, pachychoroid neovasculopathy, polypoidal choroidal vasculopathy, focal choroidal excavation, and peripapillary pachychoroid syndrome, which are referred to as pachychoroid spectrum diseases and suggest a common underlying pathogenesis [9]. Known risk factors for CSC include psychologic stress, type A personality, steroids (exogenous and endogenous), pregnancy, and sleep apnea, all of which have been implicated in the sympathetic nervous system [1]. Other factors such as hypertension, smoking, alcohol consumption, *Helicobacter pylori* infection, and several genetic mutations (*TNFRSF10A* and *GATA5*) have been reported [1]. Recent developments in ocular imaging have deepened our understanding of CSC pathogenesis. Saito and colleagues [10] used laser speckle flowgraphy to reveal choroidal hyperperfusion and choroidal blood flow imbalance in eyes with CSC. They reported that these findings could be explained by increased sympathetic activity, which causes choroidal hyperperfusion owing to increased ocular perfusion pressure caused by increased cardiac output due to systemic β-activation and choroidal blood flow imbalance due to increased vascular resistance caused by the constriction of choroidal small arterioles due to local α-activation in the eye. Recently, *en face* OCT has been reported to show characteristic findings in eyes with CSC, such as asymmetrically dilated vortex veins [11] and choroidal vascular anastomoses [12]. Widefield ICGA shows dilated vortex veins extending into the ampulla in CSC eyes, indicating that CSC involves the macular area and the entire fundus of the eye [13]. Furthermore, widefield OCT shows continuous choroidal thickening from the vortex vein ampulla to the macula, suggesting that some mechanisms cause the vortex veins to fail to drain out of the eye in eyes with CSC [14, 15]. Treatments for CSC include traditional laser photocoagulation, micropulse laser therapy, photodynamic therapy (PDT) with verteporfin, and oral administration of mineralocorticoid antagonists [1]. Recent randomized controlled trials have recommended half-dose or half-fluence PDT with laser photocoagulation limited to situations in which PDT is not available, early improvement is desired for occupational or other reasons, and the site of leakage on fluorescein angiography is outside the central fovea [1]. PDT is a valuable treatment that focuses more on the pathology because it successfully diminishes choroidal vascular hyperpermeability in the irradiated area [16], thins the pathologically thickened choroid [17], and narrows dilated large choroidal vessels [18]. Surprisingly, a decrease in choroidal thickness and choroidal vascular caliber was observed in the PDT-irradiated area and more extensively beyond the retinal vascular arcade [19, 20].

## Scleral thickness in CSC

**Fig. 1:**
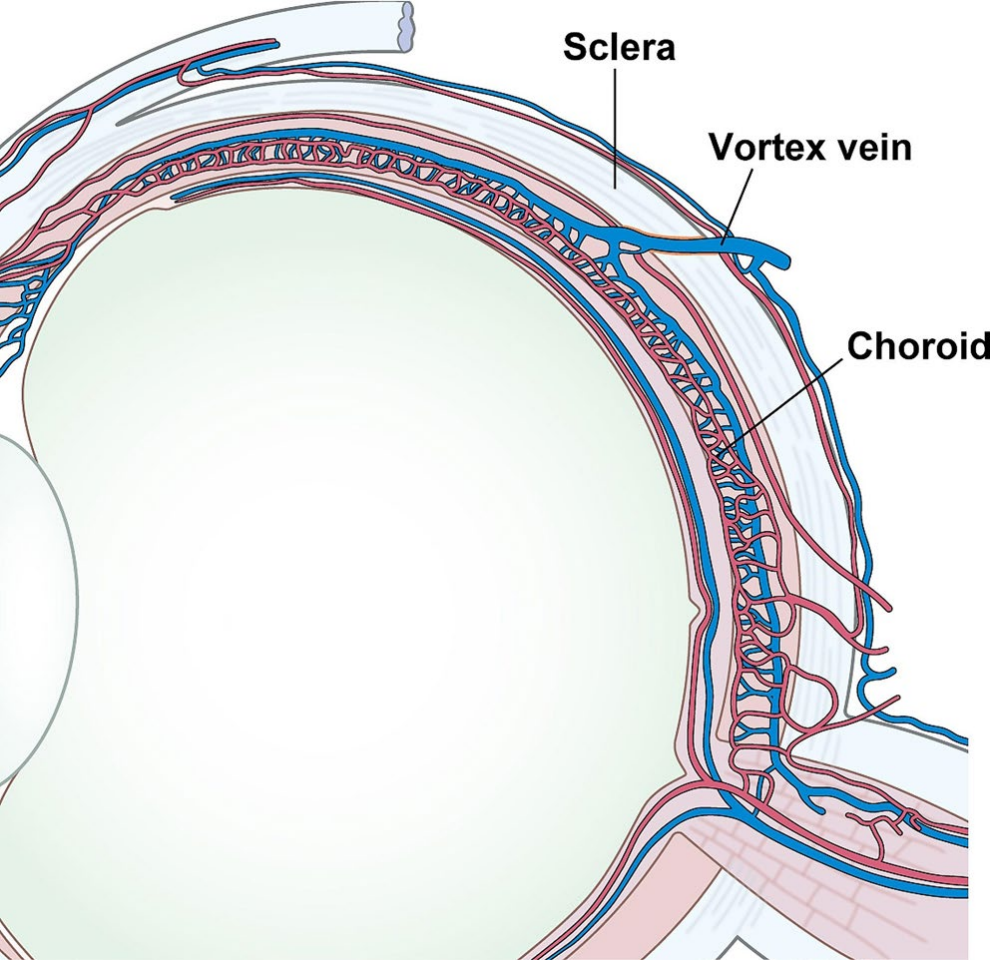
Schematic representation of the choroidal circulation. The vortex vein obliquely penetrates the sclera at the equator with a length of approximately 4 mm and drains out of the eye

**Fig. 2:**
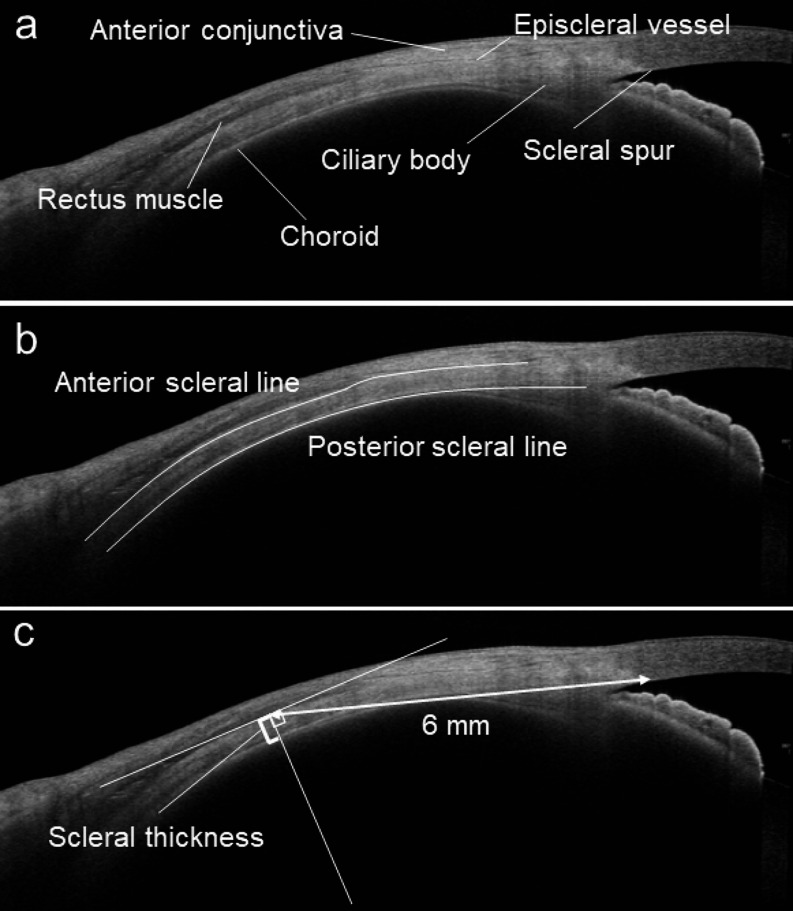
Anterior segment optical coherence tomography images used to measure scleral thickness acquired by gazing in 4 directions (superior, temporal, inferior, and nasal). **a** Scleral spur distinguished by the difference in reflectivity between the sclera and ciliary body. The episcleral vessel and rectus muscle were visualized by a low reflective line and a low reflective band, respectively. **b** Anterior and posterior scleral boundaries were determined. **c** Scleral thickness was measured vertically 6 mm posterior to the scleral spur. A line was drawn perpendicularly to a line parallel to the inner wall of the sclera, and the thickness of the sclera was measured manually. Reprinted from Imanaga and colleagues [24] with permission from Elsevier

**Fig. 3:**
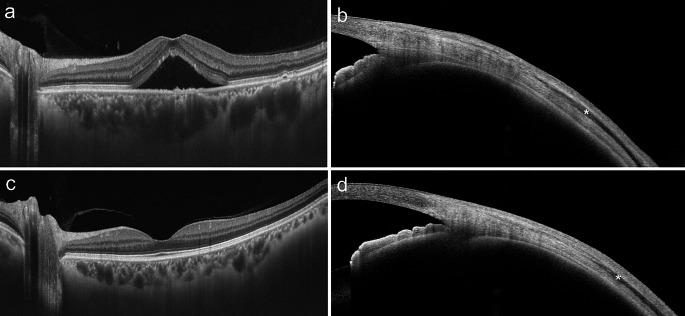
**a** Horizontal B-scan optical coherence tomography (OCT) image of a 45-year-old man with central serous chorioretinopathy showing serous retinal detachment with a subfoveal choroidal thickness of 469 μm. Significant dilation of the choroidal vessels under the fovea was observed. **b** Cross-sectional image of the temporal sclera of the same man shown in a, obtained using anterior segment OCT. The scleral thickness was 442 μm. The asterisk represents the lateral rectus muscle. **c** Horizontal B-scan swept-source OCT image of a 46-year-old man showing no significant findings with a subfoveal choroidal thickness of 320 μm. **d** Cross-sectional image of the temporal sclera of the same man shown in **c**, obtained using anterior segment OCT. The scleral thickness was 276 μm. The asterisk represents the lateral rectus muscle. Reprinted from Imanaga and colleagues [24] with permission from Elsevier

The findings mentioned above suggest an obstruction to the outflow of the vortex vein to the outside of an eye with CSC, resulting in choroidal venous congestion. Because the vortex vein in the quadrants of the choroid obliquely penetrates the sclera at the equator with a length of approximately 4 mm and drains out of the eye (Fig. 1), it is reasonable to assume that there are problems with the sclera, such as its penetration pathway. We have previously reported that the axial length of the eye is shorter and more hyperopic in CSC eyes than in normal eyes and that this tendency is more pronounced in bilateral CSC than in unilateral CSC, suggesting that ocular anatomic factors influence the pathogenesis of CSC [21]. Ideally, obtaining a cross-sectional image of the sclera and evaluating it in comparison to that of a normal eye could significantly contribute to our understanding of CSC pathogenesis. However, it is not easy to obtain cross-sectional images of the sclera with current OCT systems for the posterior region of the eye, and research has been limited to highly myopic eyes, where entire cross-sectional images of the sclera can be obtained [22]. Therefore, we attempted to acquire scleral cross-sectional images using an anterior rather than a posterior approach. However, ultrasound biomicroscopy is not suitable for accurate evaluation of scleral cross-sectional images because it is complicated: only a narrow area of the image can be obtained, the resolution is low, and above all, the sclera cannot be separated from the surrounding conjunctiva or connective tissue [23]. Therefore, we evaluated the sclera using swept-source anterior segment OCT (CASIA 2; Tomey). We found that when patients were asked to perform eye movements to obtain images under the 4 recti muscles, the recti muscles were rendered in low intensity and the sclera in high intensity by OCT, making it possible to obtain cross-sectional images of the sclera without influence from the surrounding tissue and to measure its thickness quantitatively [24] (Fig. 2). Accordingly, we compared the scleral thicknesses of 47 eyes of 40 patients with CSC with those of 53 eyes of 47 normal controls matched for age and sex, 6 mm posterior to the scleral spur. The mean scleral thickness was significantly greater in the CSC eyes than in the normal eyes in all 4 directions: the superior (429.4 μm vs. 395.2 μm; *P* = .005), temporal (447.7 μm vs. 396.5 μm; *P* < .001), inferior (455.7 μm vs. 437.8 μm; *P* = .022), and nasal (454.9 μm vs. 416.6 μm; *P* = .001) points, despite the absence of differences in spherical equivalent and axial length between the groups [24] (Fig. 3). Subsequent reports have also shown that the anterior and posterior sclera were thicker in CSC eyes than in normal eyes on anterior segment OCT [25–27] and ultrasound B-mode [28], and a consensus has been reached regarding scleral thickening in CSC eyes [29, 30]. We further investigated the separation of the luminal and stromal portions of the choroid using a binarization technique and reported that scleral thickness positively correlates with the choroidal luminal/stromal ratio in eyes with CSC [31]. In other words, thicker sclera in eyes with CSC indicates that the choroid changes to a more pachychoroid-like structure.

### Scleral thickness and suprachoroidal fluid accumulation in CSC

**Fig. 4:**
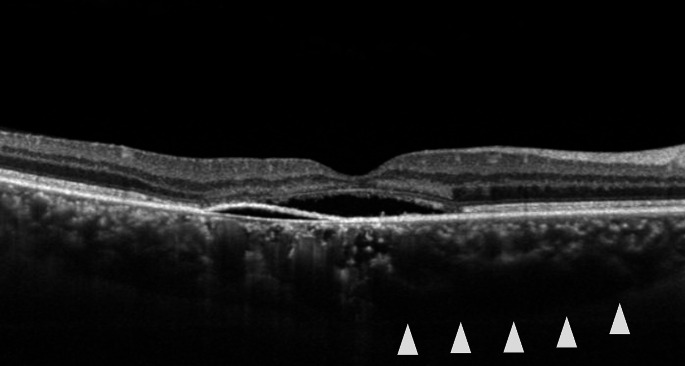
Right eye of a 51-year-old man with central serous chorioretinopathy. Horizontal B-scan with optical coherence tomography showing serous retinal detachment and pigment epithelial detachment. The subfoveal choroidal thickness was 475 μm. Dilation of the choroidal vessels under the fovea was observed. Loculation of fluid was present in the outer choroid (white arrowheads). Reprinted from Imanaga and colleagues [32]

**Fig. 5:**
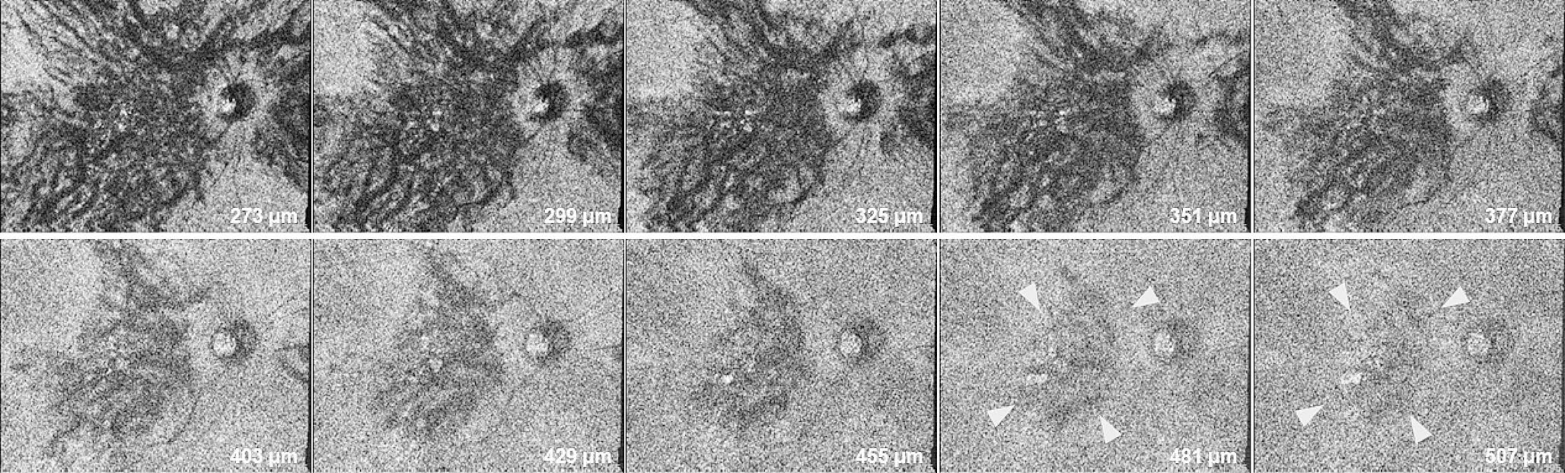
*En face* images of the case shown in Fig. 4. Volume scan data captured a 12 × 9-mm area centered at the midpoint between the foveal center and optic disc. The *en face* image was flattened at the level of the Bruch membrane. These *en face* images show the outside of the Bruch membrane from 273 to 507 μm at intervals of 26 μm. Loculation of fluid exists under the outer choroid and was confirmed to be free from connections to the horizontal and vertical choroidal vessels by use of *en face* images (white arrowheads). Reprinted from Imanaga and colleagues [32]

**Fig. 6:**
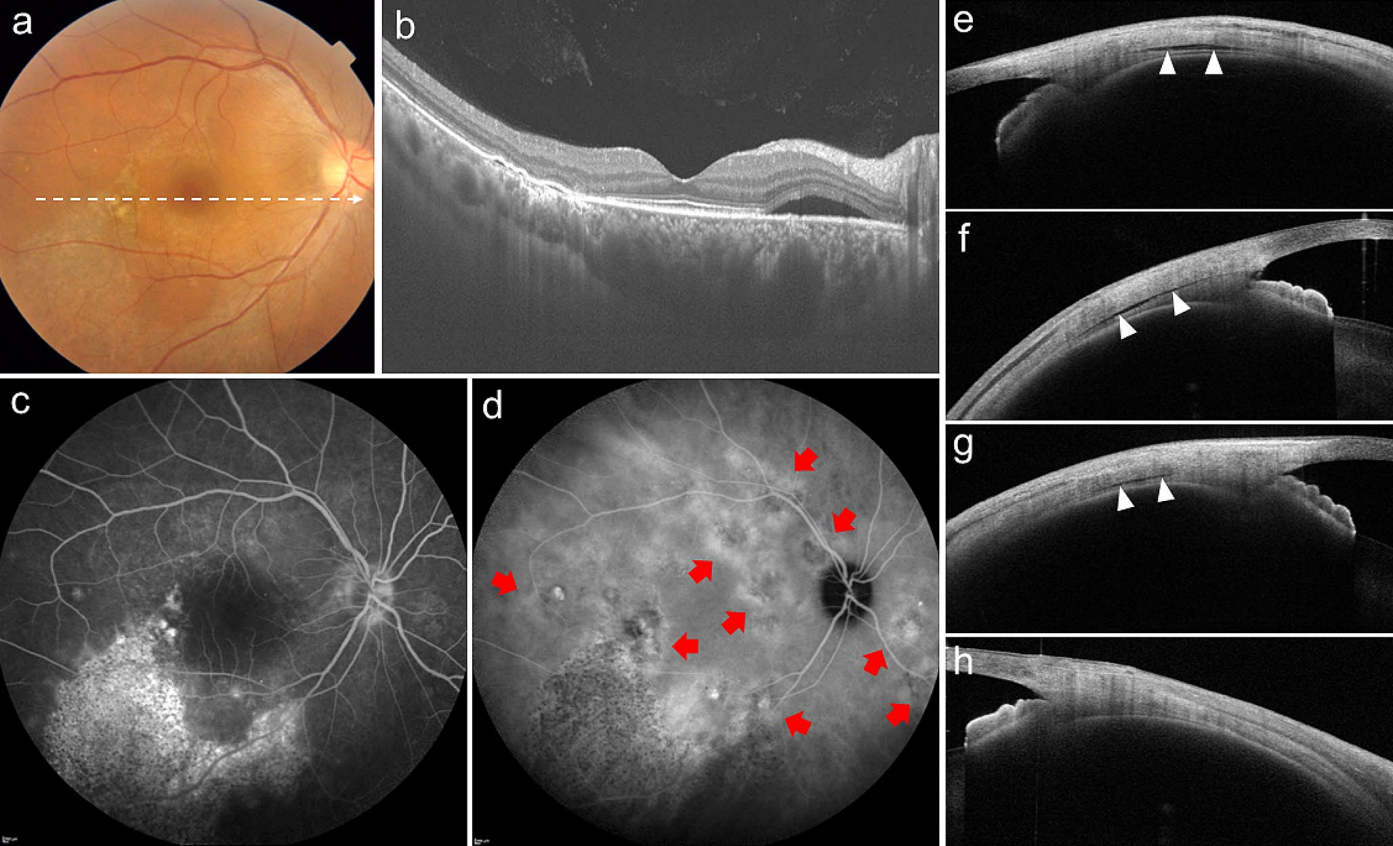
Representative case of the right eye of a 42-year-old man with central serous chorioretinopathy with ciliochoroidal effusion. **a** Color fundus photography showed subretinal fluid and a descending tract with an alteration of the retinal pigment epithelium in the macular area. **b** Optical coherence tomography (OCT) along the white dotted line in a revealed subretinal fluid and pachychoroid with markedly dilated choroidal vessels. The subfoveal choroidal thickness was 537 μm. **c** Fluorescein angiography demonstrated several leakages in the macular area. The descending tracts corresponded to the hyperfluorescent areas with window defects. **d** Indocyanine green angiography revealed multifocal areas of choroidal vascular hyperpermeability (red arrows). Anterior segment OCT demonstrated cross-sectional images of the anterior sclera in 4 directions (**e** superior, **f** temporal, **g** inferior, and **h** nasal). Ciliochoroidal effusion (arrowheads) was evident as a clearly hyporeflective area between the sclera and the ciliary body or the choroid at the superior, temporal, and inferior points. The scleral thicknesses at the superior, temporal, inferior, and nasal points were 472 μm, 534 μm, 539 μm, and 493 μm, respectively. Reprinted from Terao and colleagues [33] with permission from Wolters Kluwer Health

**Fig. 7:**
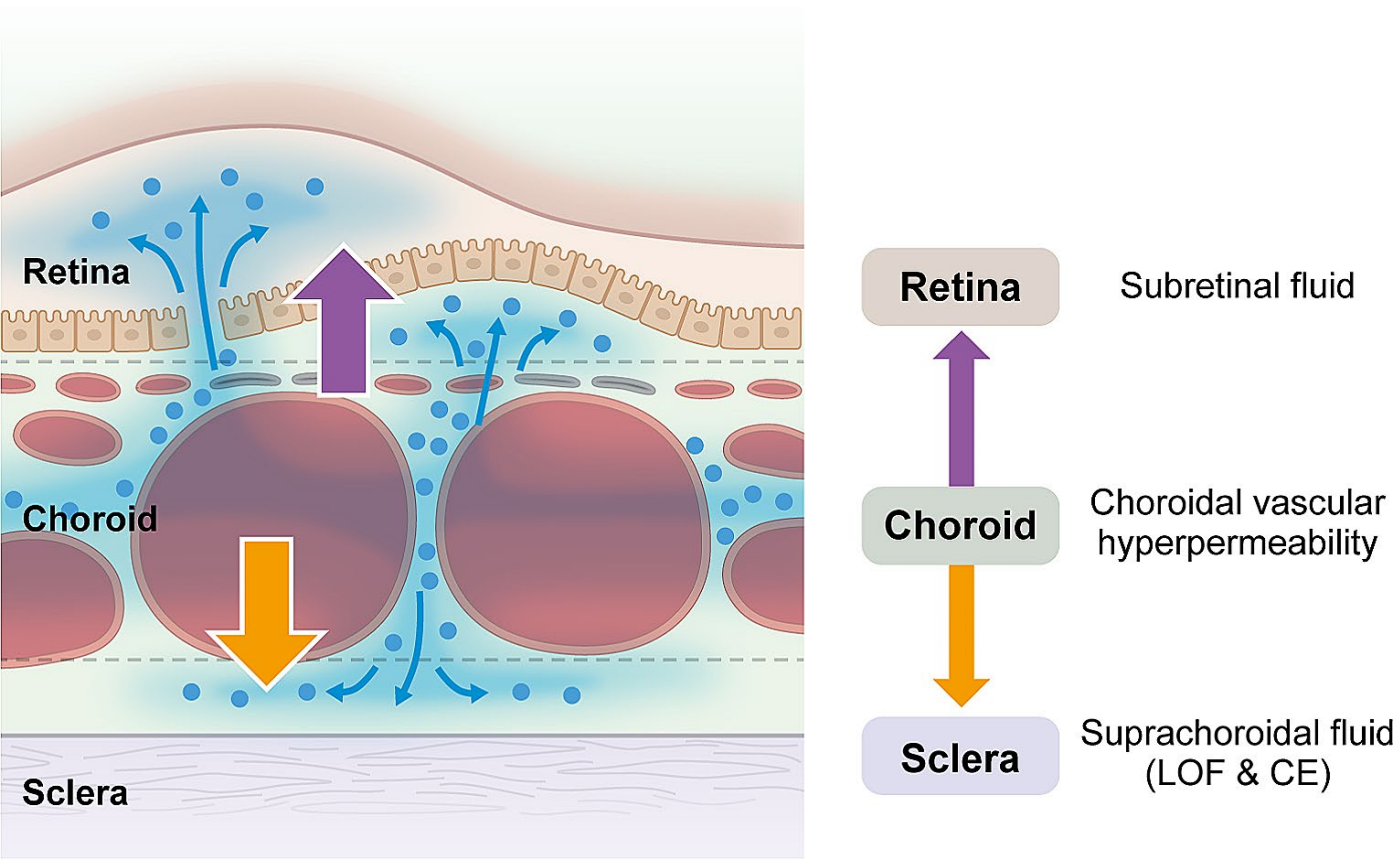
Schematic representation of fluid movement in central serous chorioretinopathy (CSC). Subretinal fluid is a characteristic feature of CSC; however, in the opposite direction, a thick sclera induces subclinical suprachoroidal fluid, such as loculation of fluid (LOF) and ciliochoroidal effusion (CE)

We further investigated the relationship between scleral thickness and suprachoroidal fluid accumulation, i.e., loculation of fluid (LOF) [32] and peripheral ciliochoroidal effusion (CE) [33], in eyes with CSC. LOF was first proposed by Spaide and Ryan in 2015 [34]. They found a high frequency of hyporeflective areas on OCT that were not choroidal vessels, which they described as fluid retention in the outer choroidal layer or suprachoroidal space. According to the same report, LOF was present in 64.8% of CSC eyes [34]; in our study, LOF was observed in 98 of 158 CSC eyes (62.0%) [32] (Figs. 4 and 5). The presence of LOF was significantly and independently associated with a thick sclera and a thick choroid. Furthermore, we observed subclinical CE using anterior segment OCT and found that the presence of CE in 1 or more of the 4 directions was significantly more common in CSC eyes (32 of 164 eyes, 19.5%) than in normal control eyes (1 of 50 eyes, 2.0%) [33] (Fig. 6). Furthermore, multivariable analysis showed that a thick sclera was the sole factor significantly associated with CE. In other words, scleral thickening may cause fluid retention in the choroid through vortex vein congestion and another pathway of decreased transscleral permeability from the intraocular to the extraocular space. In fact, the presence of CE was not associated with CVH areas, which supports this theory [33]. Subretinal fluid is usually seen as a characteristic feature of CSC in our daily practice; however, in the opposite direction, thick sclera may induce subclinical suprachoroidal fluid, such as LOF and CE (Fig. 7).

### CSC and uveal effusion syndrome (UES)

**Fig. 8:**
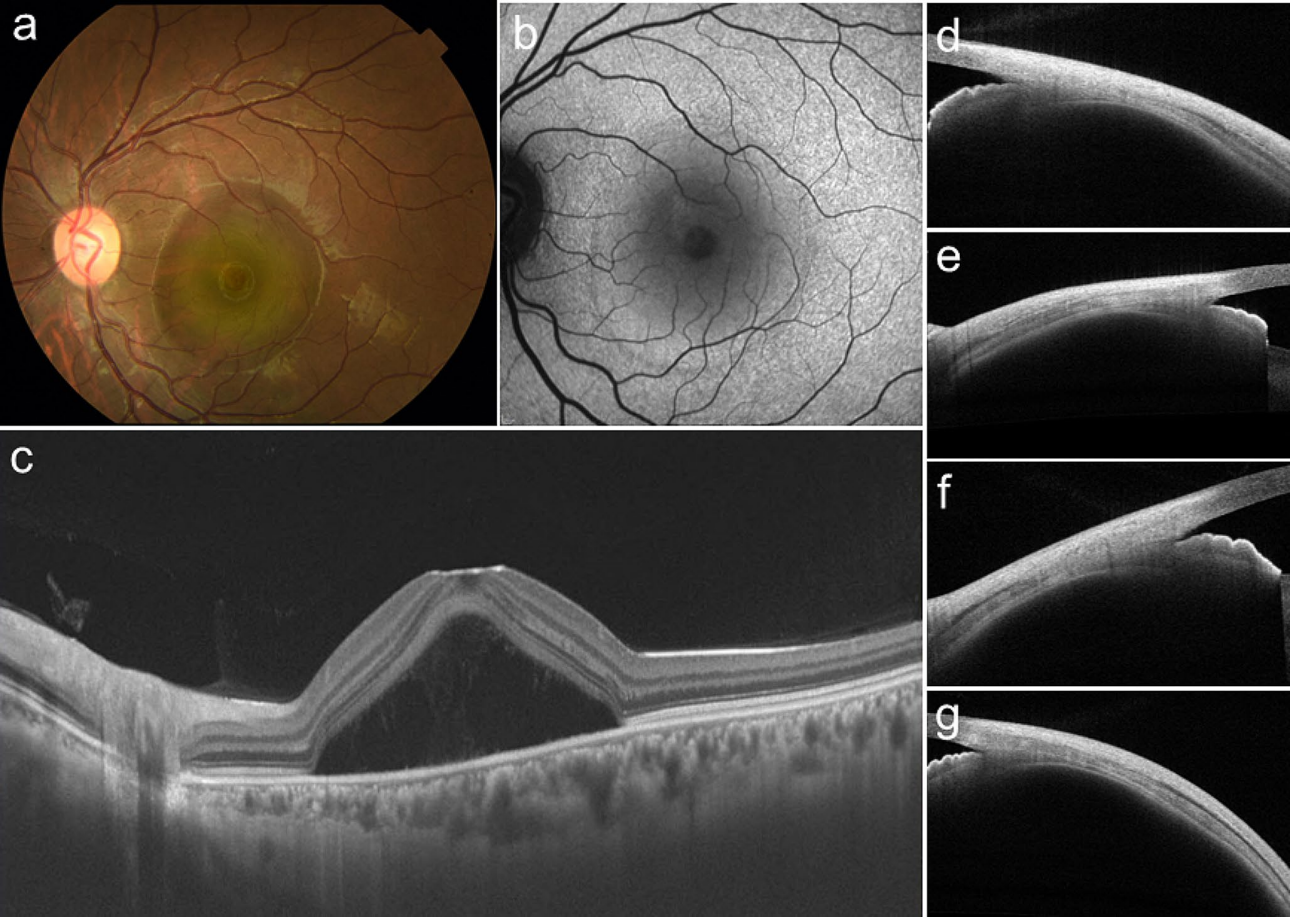
Representative case of the left eye of a 42-year-old woman with simple central serous chorioretinopathy. **a** Color fundus photography revealed serous retinal detachment in the macula. **b** Fundus autofluorescence photography demonstrated a tiny alteration of the retinal pigment epithelium. **c** Horizontal B-scan optical coherence tomography (OCT) showed high serous retinal detachment. The subfoveal choroidal thickness was 300 μm. Cross-sectional images of the sclera in 4 directions (**d** superior, **e** temporal, **f** inferior, and **g** nasal) were obtained using anterior segment OCT. The scleral thicknesses at the superior, temporal, inferior, and nasal points were 290, 290, 322, and 313 μm, respectively. Reprinted from Imanaga and colleagues [44]

**Fig. 9:**
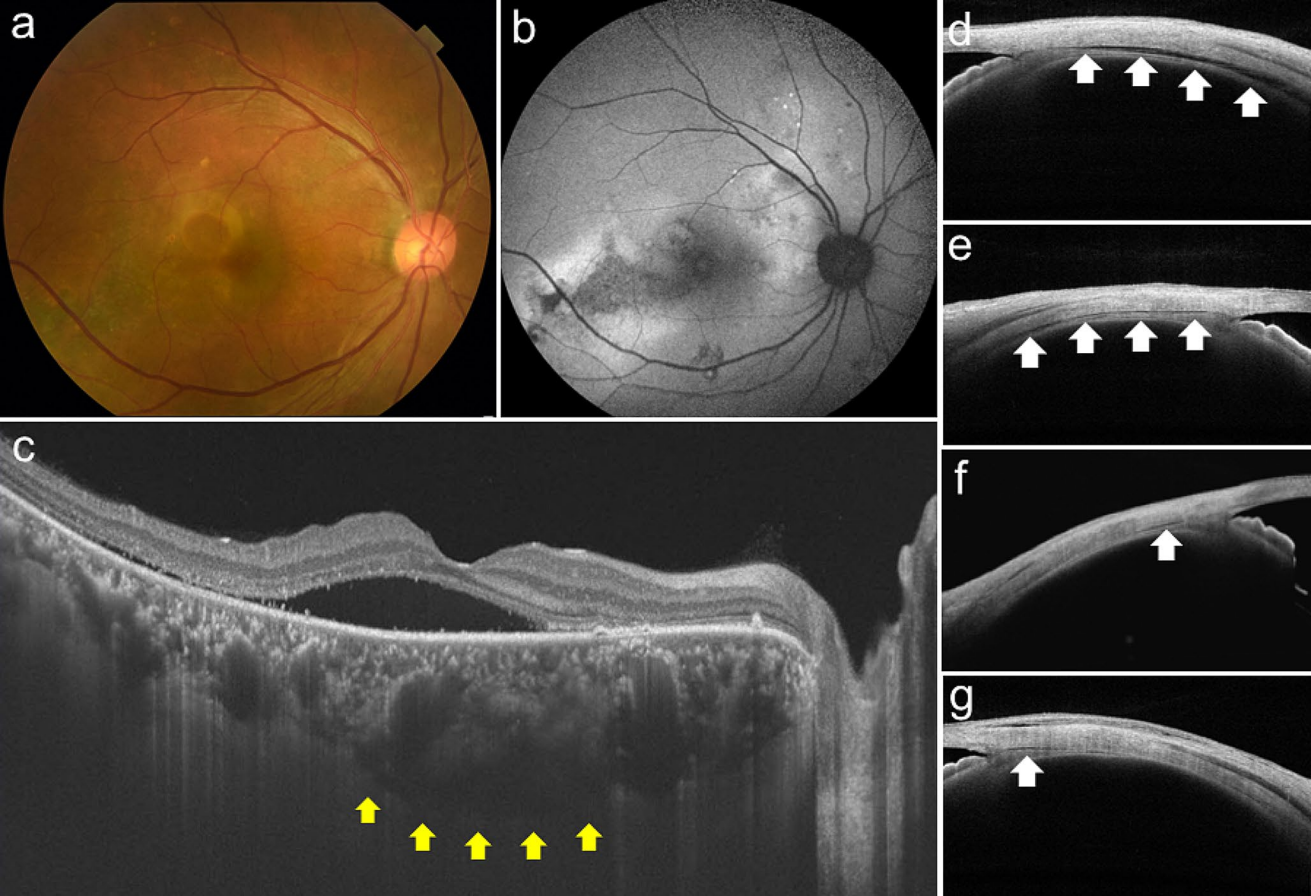
Representative case of the right eye of a 51-year-old man with complex central serous chorioretinopathy. **a** Color fundus photography revealed serous retinal detachment in the macula and retinal pigment epithelium (RPE) alteration. **b** Fundus autofluorescence photography demonstrated a total RPE atrophy area larger than 2 disc areas. **c** Horizontal B-scan optical coherence tomography (OCT) image showed serous retinal detachment and ellipsoid zone attenuation. The subfoveal choroidal thickness was 502 μm. The loculation of fluid was visualized as a hyporeflective area that was not contiguous with the vessels beneath the dilated choroidal vessels (yellow arrows). Cross-sectional images of the sclera in 4 directions (**d** superior, **e** temporal, **f** inferior, and **g** nasal) were obtained by use of anterior segment OCT. The scleral thicknesses at the superior, temporal, inferior, and nasal points were 544, 554, 581, and 572 μm, respectively. Ciliochoroidal effusion was confirmed by a hyporeflective area between the sclera and the ciliary body on anterior segment OCT images in all 4 directions (white arrows). Reprinted from Imanaga and colleagues [44]

As mentioned previously, a short axial length, hyperopia, scleral thickening, and fluid accumulation in the suprachoroidal space suggest some pathologic overlap between CSC and UES [35]. CSC is rare in highly myopic eyes [36], and some overlap exists in the clinical findings of both diseases, including choroidal circulatory disturbance [35], retinal detachment [35], and leopard spot pattern [37]. In addition, UES is not always associated with typical nanophthalmos [35]. More recently, reports of CSC complicated by typical UES findings have been published [38–40]. Additionally, sclerotomy, the standard treatment for UES, has shown an excellent response to severe CSC [41, 42], suggesting an overlap between the pathogenesis of CSC and UES. Recently, the CSC International Group proposed a classification system using multimodal imaging based on the range of retinal pigment epithelium (RPE) atrophy areas for objective evaluation of CSC [43]. We compared the scleral thicknesses of the 2 groups, namely, simple CSC (RPE atrophy ≤ 2 disc areas) and complex CSC (RPE atrophy > 2 disc areas), and found that the mean scleral thickness was greater in all 4 directions in complex CSC than in simple CSC (448.4 μm vs. 403.6 μm, 466.8 μm vs. 422.0 μm, 482.1 μm vs. 439.7 μm, 479.2 μm vs. 423.6 μm, in the superior, temporal, inferior, and nasal directions, respectively; all *P* < .001) [44] (Figs. 8 and 9). In other words, CSC and UES form the same group of “pachysclera spectrum diseases,” and complex CSC may be in a continuous position between simple CSC and UES.

## Scleral thickness in steroid-induced CSC

Steroids are one of the most important risk factors for the development of CSC [1]. Therefore, what is the involvement of the sclera in steroid-induced CSC? To answer this question, we compared scleral thickness in 96 eyes with idiopathic CSC and 14 eyes with steroid-induced CSC [45]. The results showed that the mean scleral thickness was thinner in steroid-induced CSC than in idiopathic CSC at the superior (346.6 μm vs. 423.4 μm; *P* < .001), temporal (399.4 μm vs. 440.1 μm; *P* = .020), inferior (395.3 mm v 450.1 mm; *P* = .001), and nasal (391.9 μm vs. 436.6 μm; *P* = .002) points. This result suggests that the sclera is less involved in the pathogenesis of steroid-induced CSC than of idiopathic CSC.

### Scleral thickness and vortex vein asymmetry in CSC

Asymmetrical dilation of vortex veins is a characteristic feature of the choroidal vasculature in eyes with CSC [11]. Is scleral thickening associated with vortex vein asymmetry? We examined *en face* OCT findings for factors associated with vortex vein asymmetry and found no association with scleral thickening, although a short axial length was significantly associated [46]. Interestingly, choroidal vascular asymmetry was associated with ocular anatomic factors. The asymmetric vortex veins may result in an unbalanced distribution of choroidal venous blood flow, causing congestion of certain vortex veins that may contribute to choroidal thickening and increased choroidal vascular hyperpermeability in their dominant regions [47]. Although a certain percentage of asymmetry is observed even in normal eyes [11, 48], this percentage does not increase with age, suggesting that it may be congenitally or genetically defined to some extent.

### Scleral thickness in unilateral CSC

Is scleral thickening sufficient for CSC development? CSC is often triggered by stress or exposure to steroids. To answer this question, we compared the scleral thickness of the affected and unaffected eyes of patients with unilateral CSC [49]. No differences were found in spherical equivalent, axial length, anterior chamber depth, or frequency of CE, nor in scleral thickness between the affected and unaffected fellow eyes in any of the 4 directions. The only difference between the 2 groups was in subfoveal choroidal thickness, which was significantly greater in the affected eyes than in the unaffected fellow eyes. Therefore, choroidal thickening is involved in the direct pathogenesis of CSC, and scleral thickening is an underlying factor in the development of CSC.

### Proposed pathophysiology of CSC

**Fig. 10:**
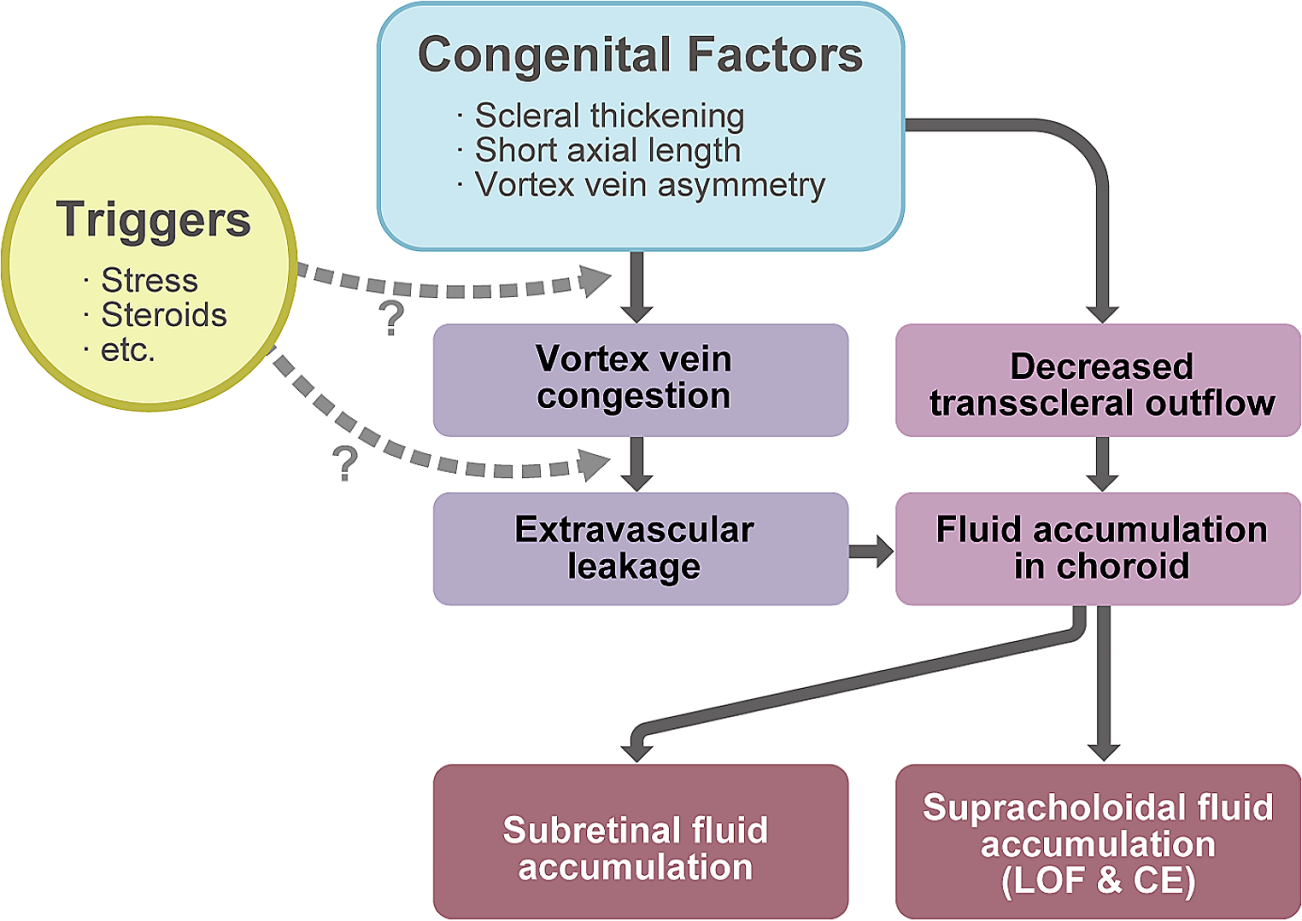
Proposed pathophysiology of central serous chorioretinopathy. First, anatomic factors, such as scleral thickening, short axial length, and vortex vein asymmetry, which may be congenital factors, seemingly cause vortex vein congestion and decreased transscleral outflow. Next, triggers, such as stress and steroids, may further induce more vortex vein congestion, extravascular leakage, and fluid accumulation in the choroid, which finally results in subretinal and suprachoroidal fluid accumulation, such as loculation of fluid (LOF) and ciliochoroidal effusion (CE)

The concept of the “two-hit theory” was recently proposed to describe the pathophysiology of CSC [50]. First, anatomic factors, such as scleral thickening [24], short axial length [21], and vortex vein asymmetry [11], which may be congenital factors, seemingly cause vortex vein congestion and decreased transscleral outflow. Next, some triggers, such as stress and steroids, may further induce more vortex vein congestion, extravascular leakage, and fluid accumulation in the choroid, resulting in subretinal and suprachoroidal fluid accumulation, although where and how these triggers work remain unclear. The proposed pathophysiology of CSC is depicted in Fig. 10.

## Future perspective

As discussed above, evidence for scleral involvement in CSC is increasing. Although evidence has been established for PDT as an effective treatment, cases of recurrence refractory to long-term treatment still exist [1]. Furthermore, the current shortage of verteporfin is a serious challenge worldwide [51], and new alternative treatments should be explored. Specifically, we would like to see the development of minimally invasive treatments targeting the sclera, especially in refractory CSC. However, our current scleral evaluation methods have limitations. First, scleral thickness measurements were performed manually. Second, we still cannot capture the site of vortex vein penetration through the sclera. Finally, only quantitative evaluations of the sclera have been conducted; qualitative evaluations will present challenges in the future. Nevertheless, apart from CSC, the pachychoroid is also suspected to be responsible for some other disorders, such as approximately 20% of dry age-related macular degeneration [52] and 50% of neovascular age-related macular degeneration [53] in the Japanese population. Detailed evaluation of the sclera in these diseases may lead to new pathophysiology, more optimal treatment, and prevention of vision-threatening conditions.
